# The challenges to control epilepsy in an isolated indigenous tribe in Brazil's Amazon: 15 years of follow-up

**DOI:** 10.1055/s-0043-1769125

**Published:** 2023-07-04

**Authors:** Marcos Manoel Honorato, Renata Carvalho Cremaschi, Fernando Morgadinho Santos Coelho

**Affiliations:** 1 Universidade Federal de São Paulo, Escola Paulista de Medicina, Departamento de Neurologia e Neurocirurgia, São Paulo SP, Brazil.; 2 Universidade do Estado do Pará, Centro de Ciências Biológicas e da Saúde, Departamento de Saúde Integrada, Santarém PR, Brazil.; 3 Universidade Federal de São Paulo, Escola Paulista de Medicina, Departamento de Psicobiologia, São Paulo SP, Brazil.

**Keywords:** Epilepsy, Seizures, Indigenous Peoples, Amazonian Ecosystem, Epilepsia, Convulsões, Povos Indígenas, Ecossistema Amazônico

## Abstract

**Background** Epilepsy is a common neurological disease that affects people all over the world, but it is rarely described in indigenous peoples.

**Objective** To study the epilepsy characteristics and risk factors for seizure control in people from an isolated indigenous population.

**Methods** This is a retrospective and historical cohort study conducted from 2003 to 2018 (15 years), at a neurology outpatient clinic, of 25 Waiwai tribes' indigenous individuals with epilepsy, inhabitants of an isolated forest reserve in the Amazon. Clinical aspects, background, comorbidities, exams, treatment, and response were studied. Factors that impacted seizure control over 24 months were identified using Kaplan-Meier curves and Cox and Weibull regression models.

**Results** The majority of cases started in childhood, with no difference regarding gender. Focal epilepsies were predominant. Most patients had tonic-clonic seizures. A quarter of them had a family history, and 20% had referred febrile seizures. There was intellectual disability in 20% of patients. Neurological examination and psychomotor development were altered in one third of the participants. The treatment controlled 72% of the patients (monotherapy in 64%). Phenobarbital was the most prescribed anti-seizure medication, followed by carbamazepine and valproate. The most relevant factors that impacted seizure control over time were abnormal neurological exam and family history.

**Conclusion** Family history and abnormal neurological exam were predicted risk factors for refractory epilepsy. Even in an isolated indigenous tribe, the partnership between the indigenous people and the multidisciplinary team ensured treatment adherence. The public healthcare system must guarantee modern anti-seizure medications, mainly for this vulnerable population, which has no other source of treatment.

## Resumo

**Antecedentes** A epilepsia é uma doença neurológica que afeta povos do mundo todo, mas raramente é descrita em povos indígenas.

**Objetivos** Estudar as características da epilepsia e os fatores de risco para o controle das crises em pessoas de uma população indígena isolada.

**Métodos** Este é um estudo de coorte retrospectivo e histórico, conduzido de 2003 a 2018 (15 anos) no ambulatório de neurologia, de 25 indígenas Waiwai com epilepsia, habitantes de uma reserva florestal na Amazônia. Aspectos clínicos, antecedentes, comorbidades, exames, tratamento e resposta foram estudados. Identificou-se os fatores que afetaram o controle das crises ao longo de 24 meses usando curvas de Kaplan-Meier e modelos de regressão de Cox e Weibull.

**Resultados** A maioria dos casos teve início na infância, sem diferença quanto ao gênero. Predominavam as epilepsias focais. A maioria dos pacientes apresentava crises tônico-clônicas. Um quarto deles tinha história familiar e 20% referiram convulsões febris. Vinte por cento dos pacientes apresentava deficiência intelectual. Um terço tinha exame neurológico e desenvolvimento psicomotor alterados. O tratamento controlou 72% dos pacientes (monoterapia em 64%). Fenobarbital foi o medicamento mais prescrito, seguido por carbamazepina e valproate, e os fatores que mais impactaram o controle das crises ao longo do tempo foram exame neurológico anormal e história familiar.

**Conclusão** História familiar e exame neurológico anormal foram fatores de risco preditores para epilepsia refratária. Mesmo em uma tribo indígena isolada, a parceria entre os indígenas e a equipe multidisciplinar garantiu a adesão ao tratamento. O sistema público de saúde deve garantir medicamentos modernos anticrise, principalmente para essa população vulnerável, que não tem outra fonte de tratamento.

## INTRODUCTION

Epilepsy is one of the most common chronic neurological diseases, affecting around 70 million people worldwide. 1 2 The biological, genetic, and environmental factors, which are associated with the etiologies of epilepsy, impact the disease and seizure control. 3 4 5

There are few studies about epilepsy in indigenous peoples. Although these have reported prevalences, such as 18.6/1,000 in the Bakairi tribe, 16/1000 in Jaguapiru Village (both in Brazil), and 57/1,000 in the Guaymi tribe (Panamá), there is a lack of follow-up of these epilepsy indigenous populations in the literature. 6 7 8 To the best of our knowledge, there is no cohort study about seizure control in isolated indigenous tribes in Brazil. 9

The present study investigates the characteristics of epilepsy among the Waiwai indigenous people, who inhabit isolated villages in a forest area on the banks of the Trombetas-Mapuera River, a tributary of the Amazon River in Brazil. 10 This study evaluates the response to epilepsy treatment during the 15-year follow-up period and identifies the risk factors associated with the seizures' refractoriness.

## METHODS

### Study design and ethical aspects

A retrospective observational study was conducted to assess epilepsy patients from the Brazilian indigenous Amazonian population named Waiwai. The historical cohort consisted of all patients with identified epilepsy who were seen at the only outpatient clinic that attended the indigenous people. The outpatient clinic, which has a multidisciplinary healthcare team, is located in Oriximiná. It is the closest village to the tribe, with 62,963 inhabitants, and the indigenous patients travel by plane for regular medical consultations. 11 After the agreement of the National Council of Ethics in Research, which is the ethical referral institution for thematic areas involving indigenous people, the tribal leadership provided written informed consent. The study was conducted at Universidade Federal de São Paulo (São Paulo, SP, Brazil). The university's Institutional Ethics Committee approved the study with the number 3.123.556.

### Study population

**Figure 1:**
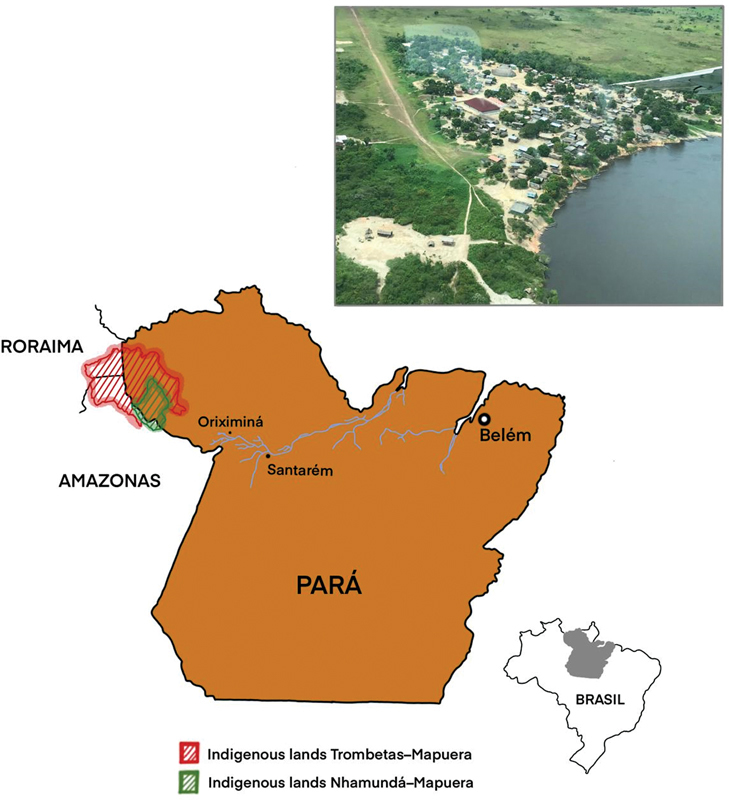
Geographic location of indigenous lands inhabited by the Waiwai indigenous.

The Waiwai indigenous people are an isolated population of ∼ 2,500 individuals, predominantly children and adults under 60. The 11 villages, which spread over 39,704 km ^2^ , do not have land access. 12 Figure 1 shows the reserve's location in the northwest region of the country, in the state of Pará. The individuals survive on family farming, extractivism, hunting, and fishing. The language spoken among them is Karib, and a minority of the population understands Portuguese. 10 We included patients diagnosed with epilepsy according to the International League Against Epilepsy (ILAE) criteria. 13 Those who had only febrile seizures or acute symptomatic seizures were excluded.

### Terms and definitions

Febrile seizures: seizures related to the occurrence of fever without central nervous system (CNS) infection, in patients between 6 months and 5 years of age. 14 Status epilepticus: prolonged seizure > 5 minutes or without recovery of consciousness if generalized motor seizures; > 10 minutes if focal, dysperceptive, or absence seizures, as well as if focal seizures > 10 minutes with impaired consciousness or > 10 to 15 minutes if absence seizures, according to the ILAE definition. 15 Abnormal neurodevelopment: delay in motor development goals (neck support, supine without support, orthostatism without support, and gait) and language development (emission of words and sentences). 16 Abnormal neurological examination: presence of alteration in one or more items of the traditional neurological examination (awareness, language, balance, gait, tonus, reflexes, sensitivity, coordination, cranial nerves, eye fundus). Perinatal hypoxia: cyanosis or Apgar < 7 at 5 minutes. 17 Intellectual disability: deficit of intellectual and adaptive abilities according to the Diagnostic and Statistical Manual of Mental Disorders, Fifth Edition (DSM-V). 18 Static encephalopathy: persistent motor deficit, posture, or tone abnormality due to non-progressive CNS damage to a developing or immature brain. 19 Response to treatment: seizure frequency after use of anti-seizure drugs. Full control: sustained absence of epileptic seizures. Partial control: reduction of up to 50% in the frequency of seizures. Refractory: reduction of less than 50% in the frequency. 20

### Data collection

Once a month, after an 8-hour boat trip, the same neurologist has been seeing patients from 2003 until now. An indigenous person trained as a health care agent translates all clinical information, frequency, and the characteristics of seizures during the consultations. The study enrolled 25 cases of epilepsy in 118 indigenous people seen at the neurology clinic between 2003 and 2018. Clinical data, family history, diagnostic exams, and pharmacotherapy were obtained in medical charts between January and June 2019. The classification of seizures and epilepsy was based on the ILAE guidelines. 21 22 All patients underwent electroencephalogram (EEG), which was reviewed by a clinical neurophysiologist during the final phase of the research. 23 Neuroimaging was possible in 64% of patients: computed tomography (CT) (40%) and magnetic resonance imaging (MRI) (24%). The patients were seen monthly for the initial 6 months. Afterwards, the appointment were held every 6 months for controlled cases.

### Statistical analyses

Sociodemographic and clinical data were examined using the IBM SPSS Statistics for Windows, version 21.0 (IBM Corp., Armonk, NY, USA). Parametric and non-parametric analyses were used in the descriptive statistics. Summary measures of numerical variables and proportions of categorical variables were calculated. Longitudinal data were analyzed using the Statistical Software R for windows, Survival Package, version 3.6.2. The results are presented as means ± standard deviation, percentages (%), and level of significance ( *p* < 0.05).

For the survival analysis procedures, two different events were possible for the outcome. (1) Control of epileptic seizures, with the time defined as the number of days from enrollment to the first occurrence of control. (2) Refractoriness or the absence of seizure control over the study time. The following parameters were used: control of epileptic seizures as an event; observation period of 24 months from medical consultation onset (t = 0); KM curves to estimate the influence of each independent variable; Log-rank (LR), Gehan-Breslow (GB) and Tarone-Ware (TW) tests; Cox regression, and Weibull models.

## RESULTS

Twenty-five cases of epilepsy were diagnosed during the 15 consecutive years of follow-up, corresponding to 21.2% of all 118 indigenous patients seen at the neurological clinic. The median onset age of epilepsy was 2 years, between less than 1 month to 77 years. At the initial medical consultation, 44% of the patients reported uncountable seizures, and 28% had only 2 episodes. Only 20% of the patients had received any previous treatment.

Table 1 summarizes the demographic and pathological characteristics of epilepsy in the Waiwai indigenous people, with all the predictors' variables involved in controlling the crises. Twelve patients were male (48%). The mean onset age of epilepsy was 10.2 ± 16.2 years, and epilepsy started predominantly in childhood (68%). A family history of epilepsy, consanguineous parents or personal history of febrile seizures or status epilepticus, occurred in 24% of the sample. Psychomotor development delay (32%), abnormal neurological examination (28%), perinatal hypoxia (20%), intellectual disability (20%), and static encephalopathy (12%) were identified. Generalized seizures (56%) predominated. Abnormalities in EEG were seen in 68% of the patients, and 24% of them had pathological neuroimaging. Focal epilepsies were more frequent than generalized (64% versus 36%). The mean pharmacological treatment follow-up time was 5.2 ± 3.4 years. Most patients used monotherapy (64%), principally phenobarbital (40%). Those in polytherapy essentially used the association of carbamazepine with clobazam (16%). The control of seizures was complete in 40% and partial in 32% of the patients.

**Table 1:** Demographic and pathological characteristics of epilepsy in the Brazil's Amazon indigenous Waiwai tribe

Variable		Epilepsy ( *n* = 25)	Description	*P* -value
Gender male		12	48%	0.8
Onset age, years (mean)			10.2 ± 16.2 SD	
Age category	Children	17	68%	**0.001**
	Teen	4	16%	
	Adults and elderly	4	16%	
Family history of epilepsy		6	24%	
Consanguineous parents		6	24%	
Febrile seizures		6	24%	
Status epilepticus		6	24%	
Abnormal neurodevelopment		8	32%	
Abnormal neurological examination		7	28%	
Perinatal hypoxia		5	20%	
Intellectual disability		5	20%	
Static encephalopathy		3	12%	
Generalized seizures		14	56%	
Abnormal electroencephalogram		17	68%	
Abnormal neuroimaging		6	24%	
Epilepsy classification	Focal	16	64%	0.08
	*Unknown etiology*	9	36%	
	*Structural*	5	20%	
	*Probably genetic*	2	8%	
	Generalized	9	36%	
	*Probably genetic*	7	28%	
	*Structural*	2	8%	
Follow-up time (mean, years)			5.2 ± 3.4 SD	
Treatment	Monotherapy	16	64%	0.08
	*Phenobarbital*	10	40%	
	*Carbamazepine*	3	12%	
	*Valproic acid*	3	12%	
	Polytherapy	9	36%	
	*Carbamazepine + Clobazam*	4	16%	
	*Valproic acid + topiramate + clobazam*	2	8%	
	*Others*	3	12%	
Response to treatment	Full control	10	40%	0.02
	Partial control	8	32%	
	Refractoriness	7	28%	

Abbreviation: SD, standard deviation.

Note: significant *p* -values in bold.

The Indigenous Health District responsible for assisting the Waiwai people provides the anti-seizure medications free of charge: phenobarbital, carbamazepine, phenytoin, valproic acid, while topiramate, clobazam, and oxcarbazepine can be purchased on demand (special purchase order). The multidisciplinary team controls the supply, administration, and side effects of the drugs, and they reported that only 1 patient (4%) refused the treatment.

**Figure 2:**
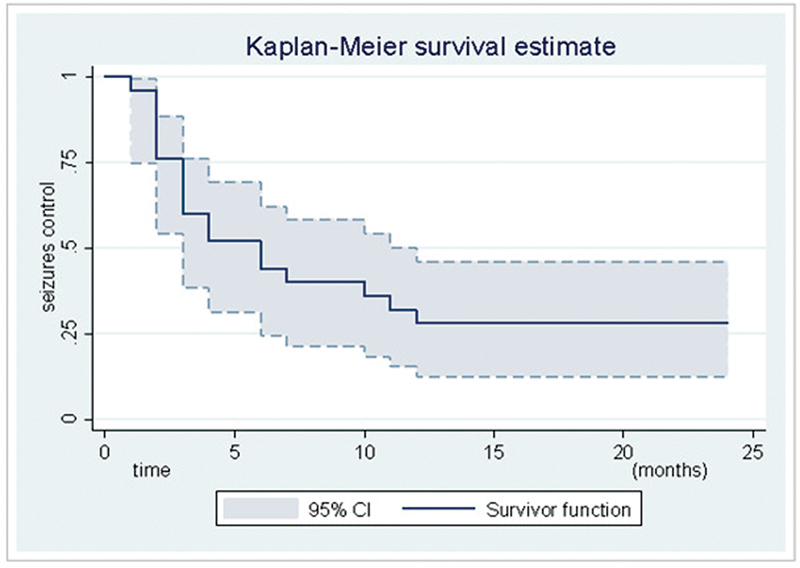
Curve and 95% confidence intervals from the Kaplan-Meier estimator for seizure control.

**Figure 3:**
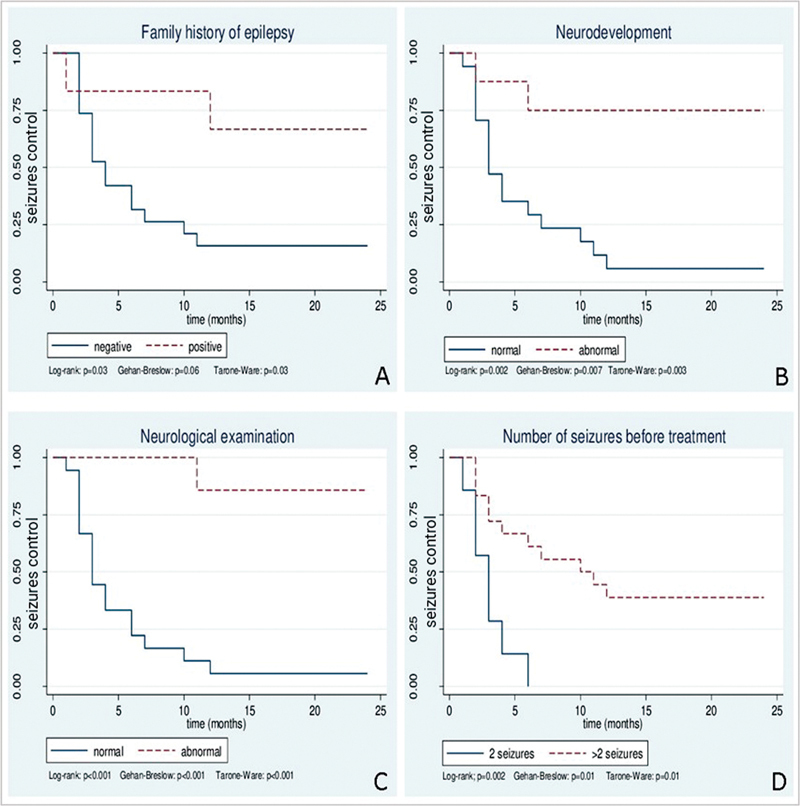
Curve estimated by Kaplan-Meier and statistical tests for seizures control in the variables family history of epilepsy (A), neurodevelopment (B), neurological examination (C), number of seizures before treatment (D).

Figure 2 shows the Kaplan-Meier (KM) estimate curves for seizure control. Figure 3 demonstrates the KM estimate curves for seizure control of the 4 independent variables statistically significant for the outcome: family history of epilepsy (GB = 0.06; TW = 0.03), neurodevelopment (GB = 0.007; TW = 0.003), neurological examination (GB < 0.001; TW < 0.001), and number of seizures before treatment (GB = 0.01; TW = 0.01).

In Table 2 , the Log-rank test evidenced that the variables positive family history of epilepsy ( *p* = 0.03), abnormal neurodevelopment *(p = 0* .002), abnormal neurological examination ( *p* = 0.001), and more than 2 seizures before treatment ( *p* = 0.002) were significant for a longer time to control the crises. The abnormal neurological examination had the highest HRs (Hazard Ratio) (15.4 versus 14.1), which represents a greater risk of refractoriness.

**Table 2:** Survival Analysis: Statistical correlation between each independent variable and the outcome control of seizures, using the Log-rank test in Kaplan-Meier estimates

Independent variable	*P-* value	Hazard ratio
Family history of epilepsy	**0.03**	4.6
Abnormal neurodevelopment	**0.002**	9.8
Abnormal neurological exam	**0.001**	14.1
Early onset ^a^	0.7	–
> 2 seizures before treatment	**0.002**	9.4
Intellectual disability	0.06	–
Cerebral palsy b	0.09	–
Febrile seizure	0.99	–
**Epilepsy classification**	**0.2**	–

Notes: ^a^ < 3 years old.

b static encephalopathy; significant p values in bold.

**Figure 4:**
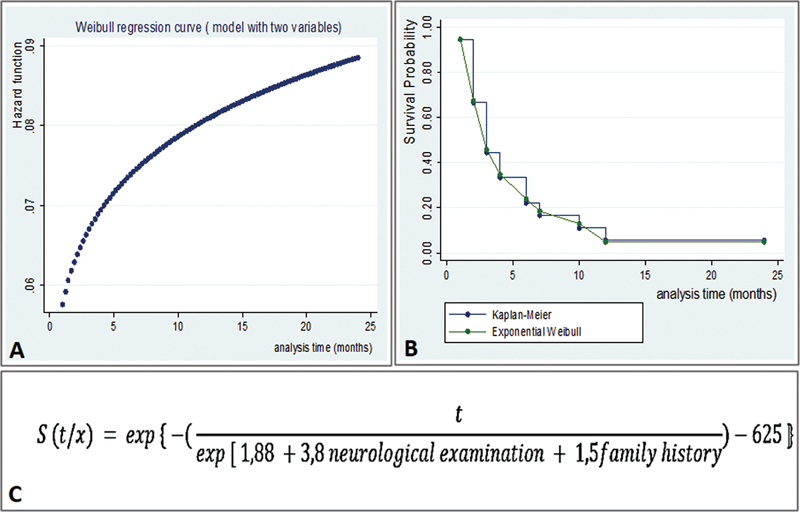
Abbreviations: S, survival; t, time; exp, exponential. Analysis of seizures control time by Weibull's model with two variables: exponential curve generated (A), residual and Kaplan-Meier estimated curves compared (B) and survival function (C).

The Weibull model fitted for 2 variables (AIC = 65.3) got *p* = 0.001 and time ratio ratio = 17 in the abnormal neurological examination; and time ratio = 1.5 in the family history of epilepsy (95% confidence intervals 2.37–122.49 and 0.4–6.13, respectively). The generated curves and equation can be seen in Figure 4 . Models with a larger number of variables did not fit, probably due to the reduced sample size.

## DISCUSSION

Our study is the first study with a follow-up of epilepsy patients in an isolated indigenous tribe in Brazil. The absence of gender predominance in our studied population had already been reported. 24 The Waiwai tribe's age pyramid contributes to the age onset prevalence. The mean and median onset age of the Waiwai were lower than those of the Bakairi, Jaguapiru, and Guaymi tribes. 6 7 8 The rare cases of epilepsy in adulthood in the neurological clinic were probably associated with the uncommon, acquired causes of the disease in the geographically isolated Waiwai tribe, where violence is minimal, and traffic accidents are absent, as well as traumatic brain injury, and cerebrovascular diseases. 25 26

The prevalence of febrile seizures in our Waiwai indigenous sample was higher than in non-indigenous populations and lower than in non-isolated indigenous people, such as the Guaymi (44%). 7 27

Perinatal hypoxia, psychomotor development abnormalities, and related comorbidities did not differ from the general epileptic population. 28 29 The frequency of abnormal neurological examination, mainly in children, was similar to the one reported in the literature, mostly in children or elders. 30 The predominance of generalized seizures was lower than the one of the Guaymi tribe (53%) and similar to focal crises in the Bakairi tribe (55%). 6 7

We found a high percentage of abnormal EEG compared with the Bakairi tribe (11%). 6 Some Waiwai patients could not undergo neuroimaging studies due to their remote geographic location. Most of these tests performed were normal, which is usually seen. 31

There was a predominance of focal epilepsies with an unknown etiology. Idiopathic and cryptogenic were the most frequent in this study. Likewise, the data from a metanalysis and the urban area of São José do Rio Preto (Brazil), a medium size city. 32 33

All patients in this study were on anti-seizure medication, which differs from what was observed among the Bakairi indigenous, among whom only a third were using pharmacotherapy. 6 Facing cultural differences, the indigenous people rarely accept the use of continuous medication. The tribes throughout Latin America give a mystical interpretation of the disease and often use medicinal plants and rituals to try to control the symptoms. The treatment adherence was good in our sample, probably due to the constant intervention of the indigenous multidisciplinary team who administered the medicines. 6 34 35

Phenobarbital was the most prescribed anti-seizure medication, followed by carbamazepine and valproate. These drugs were the first-line pharmacological option due to their availability in our public healthcare system. There was a higher probability of seizure control up to 6 months after the anti-seizure medication onset, and it became lower between 6 and 24 months. The unavailability of new-generation anti-seizure medications can be implicated, mainly in this population. Brazil's public health care system has a limited number of existing medicines, and there is no other access to treatment. 36

Status epilepticus occurred in a quarter of Waiwai patients, mainly in children, similar to what is reported in worldwide registries. 37 This severe complication of epilepsy generally affects the male gender, black ethnicity, and children, often due to the interruption of pharmacotherapy. 37 Even though epilepsy causes premature death in low- and middle-income countries, it was not a significant factor in mortality among our sample of Waiwai indigenous. 38 The only death occurred in a patient who had refractory focal epilepsy since the age of 33 years old, but it was associated with chronic myeloid leukemia at the age of 60.

Our findings revealed that family history of epilepsy, abnormal neurological examination, neurodevelopment abnormality, and number of seizures before treatment impacted seizure control in our sample. These findings are in accordance with the risk factors for the control of epilepsy described in the literature. 2 36 39 40 In this sample of Waiwai indigenous patients, neither the classification of epilepsy nor the age at epilepsy onset influenced the control of the disease over time. Non-indigenous people with focal epilepsies and early onset are more susceptible to refractoriness. 2 39 40 41

The most relevant risk factors to increase the risk of failure seizure control over time in the studied population were positive family history and abnormal neurological exam. It is in agreement with the literature on non-indigenous people. 40 41

The present study had some limitations that should be mentioned. In this study, it was impossible to determine the prevalence of epilepsy among the Waiwai indigenous. The door-to-door data collection was not feasible, facing the sizeable territorial extension of the reserve. As many patients did not have access to neuroimaging due to geographic and healthcare system difficulties, analyzing this variable in controlling the crises was impossible. The patients with focal epilepsy of unknown etiology could have had another classification if they had brain imaging. The unavailability of newer anti-seizure medications may have influenced seizure control. The small sample size may have influenced the analysis of the classification of epilepsy and the age of disease onset.

This was the first study with a follow-up of epilepsy patients in an isolated indigenous tribe in Brazil. Treatment adherence can be possible in this population with constant intervention and partnership between the indigenous people and the multidisciplinary team. Family history and abnormal neurological exam were predicted risk factors for refractory epilepsy, and the public healthcare system should guarantee access to modern medications anti-seizure for these vulnerable populations.
